# Self-medication by orang-utans (*Pongo pygmaeus*) using bioactive properties of *Dracaena cantleyi*

**DOI:** 10.1038/s41598-017-16621-w

**Published:** 2017-11-30

**Authors:** H. C. Morrogh-Bernard, I. Foitová, Z. Yeen, P. Wilkin, R. de Martin, L. Rárová, K. Doležal, W. Nurcahyo, M. Olšanský

**Affiliations:** 1 The Orang-utan Tropical Peatland Project (OuTrop), Palangkaraya, Central Kalimantan Indonesia; 2 Centre for Ecology & Conservation, College of Life and Environmental Sciences University of Exeter, Penryn Campus, Penryn, Cornwall TR10 9FE United Kingdom; 3 Department of Botany and Zoology, Masaryk University, Kotlářská 2, 611 37 Brno, Czech Republic; 4 The Centre for International Cooperation in Sustainable Management of Tropical Peatlands (CIMTROP), University of Palangka Raya, Central Kalimantan, Indonesia; 5 Royal Botanic Gardens, Kew, Richmond, Surrey, UK; 6 Department of Vascular Biology and Thrombosis Research, Medical University of Vienna, A-1090 Vienna, Austria; 7 Department of Chemical Biology and Genetics & Laboratory of Growth Regulators, Centre of the Region Haná for Biotechnological and Agricultural Research, Faculty of Science, Palacký University and Institute of Experimental Botany, Academy of Sciences of Czech Republic, 78371 Olomouc-Holice, Czech Republic; 8 Department of Parasitology, Faculty of Veterinary Medicine, Gadjah Mada University, Yogyakarta, Indonesia; 9 Foundation UMI-Saving of Pongidae, Brno, Czech Republic

## Abstract

Animals self-medicate using a variety of plant and arthropod secondary metabolites by either ingesting them or anointing them to their fur or skin apparently to repel ectoparasites and treat skin diseases. In this respect, much attention has been focused on primates. Direct evidence for self-medication among the great apes has been limited to Africa. Here we document self-medication in the only Asian great ape, orang-utans (*Pongo pygmaeus*), and for the first time, to our knowledge, the external application of an anti-inflammatory agent in animals. The use of leaf extracts from *Dracaena cantleyi* by orang-utan has been observed on several occasions; rubbing a foamy mixture of saliva and leaf onto specific parts of the body. Interestingly, the local indigenous human population also use a poultice of these leaves for the relief of body pains. We present pharmacological analyses of the leaf extracts from this species, showing that they inhibit TNFα-induced inflammatory cytokine production (E-selectin, ICAM-1, VCAM-1 and IL-6). This validates the topical anti-inflammatory properties of this plant and provides a possible function for its use by orang-utans. This is the first evidence for the deliberate external application of substances with demonstrated bioactive potential for self-medication in great apes.

## Introduction

There are many documented examples of how animals, ranging from butterflies, caterpillar larvae and birds self-medicate and why they do it^1–3^. The first suggested possibility for the use of secondary plant metabolites as anthelmintics, laxatives, antibiotics, and/or antidotes after toxin consumption was described in 1981^4^. Since then animals have been documented to use a variety of minerals, plant parts, and arthropods by ingesting them or anointing them to their fur/hair or skin (‘fur-rubbing’) in order to treat internal and external parasites, ailments, and skin infections^5–9^. Some of the most thoroughly documented accounts of self-medication are of primate species, particularly African great apes: chimpanzees (*Pan troglodytes troglodytes, P. t. schweinfurthii*, *P*. *t*. *verus*, *P*. *t. velerosus*), bonobos (*Pan paniscus*) and gorillas (*Gorilla gorilla gorilla* and *G. g. graueri*)^10–15^. In all these cases, the method of ingestion was similar, with individuals chewing the pith and swallowing the bitter juice or folding up and swallowing whole hairy leaves in order to control intestinal nematode and cestode infections. To date, only one Asian primate, the white-handed gibbon, a Lesser ape, has been reported to engage in leaf swallowing in Thailand, resulting in the expulsion of parasites^16^. While there is yet no report concerning primary self-medication (the ingestion of medicative substances) in the orang-utans (*Pongo abelii, P. pygmaeus* – the only Asian great apes), a number of plants with potential medicinal value have been recorded in their diet, some of which are also used in traditional human medicine^17^. It has also been shown that the prevalence of several plant species in the diet of orang-utans correlates directly with the presence of parasites in individuals, as opposed to, their prevalence in the environment^18^.

The first and only report so far of possible secondary self-medication (the external application of medicinal substance) in orang-utans, is in the Bornean orang-utan^19^. Our behavioural observations of these habituated wild orang-utans began in September 2003 and over the course of a 10-year period, we collected over 20,000 hours of observational data on 50 individuals. Fur-rubbing with *Dracaena cantleyi* Baker was observed on only seven occasions by six different females (all but one had offspring) and one flanged male. This plant was previously misidentified as *Commelina* sp. in 2008^12^. In each case, the apical parts of leaves were bitten off and chewed into a pulp, releasing saponins to produce a white soapy lather. This lather was then rubbed onto either the upper arms or upper legs for a length of time between 15 and 45 minutes. This action was methodical and purposeful. None of the leaf was swallowed and the remaining chewed pulp was always spat out. This was the first account of secondary self-medication in apes of either Asia or Africa, as all previous reports on fur-rubbing have come from several Neotropical primates and one species of lemur^3,5,20^. In these species, plant materials were applied either directly to the skin or chewed and then rubbed into the fur for purposes including ectoparasite removal, insect repellent, treating fungal or bacterial skin infections, treating wounds, or for soothing, stimulating, or conditioning the skin or fur^6–9,21–23^.

Aim of this paper is to determine the biological properties of *Dracaena cantleyi* to test our ideas on the possible function of fur-rubbing in the Bornean orangutan.

## Results

### Biological activities of *Dracaena cantleyi*

#### Extracts from *D. cantleyi* inhibited expression of inflammatory cell adhesion molecule E-selectin (ELAM, CD62E)

**Figure 1:**
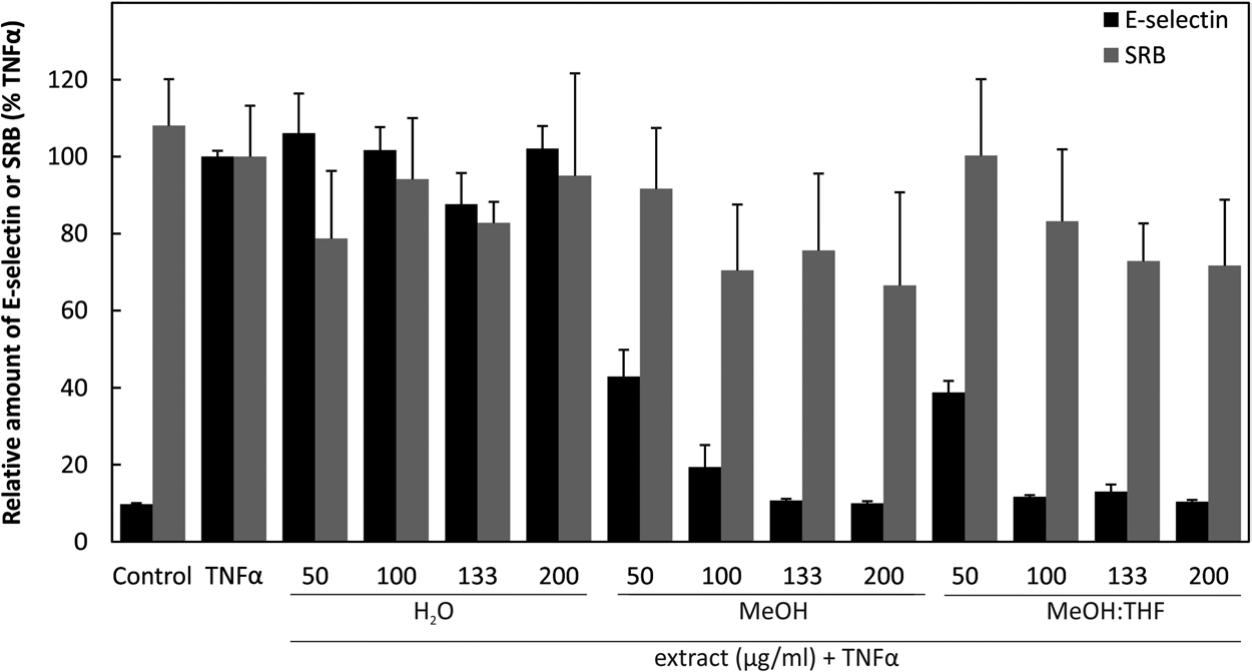
Cell surface ELISA for cell adhesion molecule ELAM. HUVECs were pre-treated with the different extracts from *D. cantleyi* (50, 100, 133 and 200 µg/ml as indicated) for 30 min followed by TNFα stimulation for 4 h. Viability was determined by the sulforhodamine B cytotoxicity assay (SRB).

Confluent Human Umbilical Vein Endothelial Cells (HUVEC) were pre-stimulated with the different extracts as indicated for 30 minutes and then exposed to the inflammatory cytokine TNFα (Tumor Necrosis Factor α) for 4 hours to stimulate NFκB (Nuclear Factor Kappa-Light-Chain-Enhancer of Activated B Cells), and thus E-selectin CD62E. Subsequently, the level of ELAM protein was determined using cell surface ELISA (Enzyme-Linked Immunosorbent Assay). Following this, the treatment cells still remained viable as determined by the Sulforhodamine B Cytotoxicity Assay SRB assay, but the level of ELAM decreased in a dose-dependent manner for methanol (MeOH) extract and methanol:tetrahydrofuran MeOH:THF extract (Fig. 1).

#### Expression of the inflammatory cytokine interleukin-6 (IL-6) decreased by treatment with methanol extract of *D. cantleyi*

**Figure 2:**
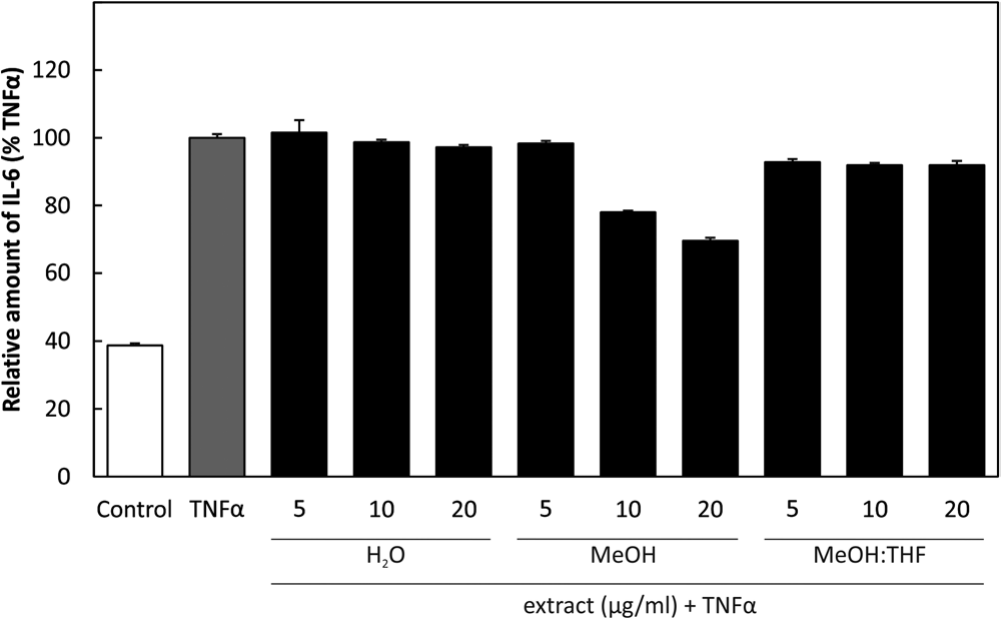
IL-6 ELISA: A sandwich ELISA was used to determine IL-6 production by HUVEC in the cultivation medium after pre-stimulation with 5, 10 and 20 µg/ml of extracts for 30 min and activating with TNFα for 24 hours.

The levels of inflammatory cytokine IL-6 were measured by sandwich ELISA in the cultivation medium from HUVEC pre-stimulated with the different extracts as indicated, for 30 minutes and then exposed to TNFα for 24 hours. A dose-dependent reduction was observed by 10 and 20 µg/ml of methanol extract (Fig. 2).

#### Extracts from *D. cantleyi* reduced cell surface expression of ICAM-1 on HUVEC

**Figure 3:**
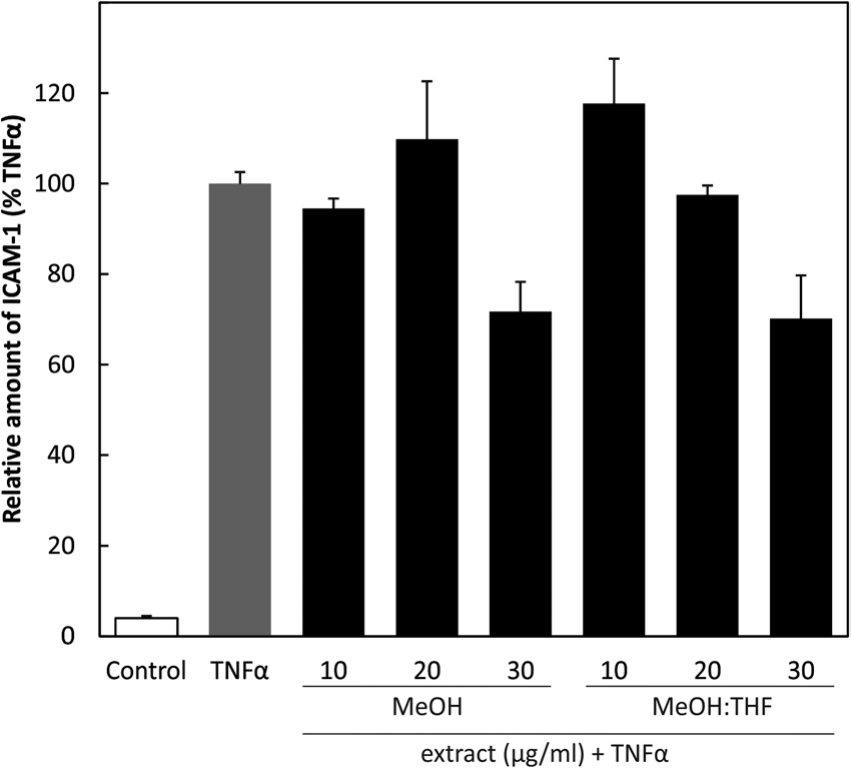
ICAM-1 ELISA: HUVECs were treated with 10, 20 and 30 µg/ml of extracts for 30 min followed by TNFα for 24 h and ICAM-1 determined by sandwich ELISA.

One of the genes regulated by NFκB and down-regulated by the extracts tested is intercellular adhesion molecule-1 (ICAM-1). Expression of ICAM-1 contributes to cell adhesion or invasiveness. We therefore examined the effects of extract treatment on ICAM-1 expression in HUVECs, by pre-treatment of the cells for 30 minutes, and then with TNFα for 24 hours. Expression of ICAM-1 was determined by sandwich ELISA. Both the methanol and methanol-tetrahydrofuran extracts reduced ICAM-1 expression dose-dependently, with an approximate IC_50_ value of 30 µg/ml (Fig. 3).

#### Vascular cell adhesion molecule (VCAM-1) expression is decreased by treatment with *D. cantleyi* extract

**Figure 4:**
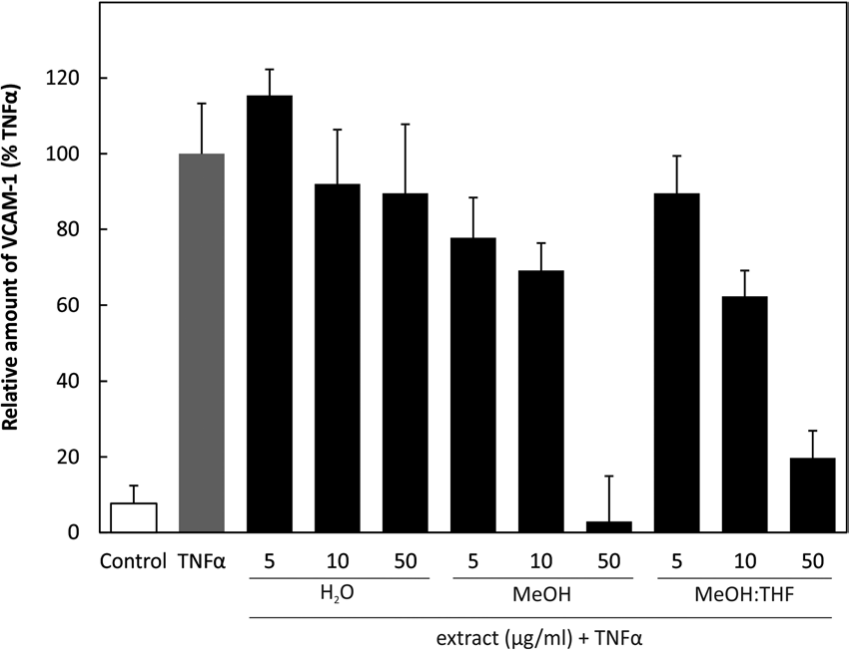
Flow cytometry VCAM-1: HUVEC were pre-stimulated with 5, 10 and 50 µg/ml of extracts for 30 min as indicated and then by TNFα for 24 hours. The level of VCAM-1 was determined using flow cytometer.

Flow cytometric analysis showed decrease of the expression of VCAM-1 in HUVEC cells after the 30-minute pre-treatment with extracts and activating with TNFα for 24 hours. Expression of (VCAM-1) is known to be regulated by NFκB. Methanol and methanol-tetrahydrofuran extracts inhibited dose-dependently (5, 10, 50 µg/ml) the level of VCAM-1 (Fig. 4).

## Discussion

Since our first observations of fur-rubbing with *D. cantleyi* by Bornean orang-utans in the Sabangau Peat-swamp forest in Central Kalimantan province, Indonesia in 2003, we have documented a total of ten cases up to September 2015. This is a very rare behaviour. To our knowledge, this is the only place in the range of either *Pongo* species where this behaviour has been witnessed. Chemical analyses of the properties of this plant are consistent with the hypothesis that fur-rubbing is a form of self-medication used to treat joint and muscle inflammation^19^. It is also important to note that local indigenous human inhabitants use this plant for the same purpose. This is also the case for use of *Vernonia amygdalina* by chimpanzees in Western Tanzania, where the indigenous human population also uses this plant for the treatment of parasites and stomach upset; recovering from similar symptoms in roughly the same amount of time^10,12^.

The secondary metabolite saponin present in the leaves generates foam when in an aqueous solution. This allows the orang-utan to create a soapy lather by chewing the leaves, making it perhaps easier to rub the compounds onto the affected body part. Saponins are found in many flowering plants, but are especially abundant in monocotyledons^24,25^. These compounds are most abundant in *Dioscoreales*, *Asparagales* and some taxa of *Liliales* (in the sense of APG3, 2009), but saponins are toxic to some animals, and tend to be unpalatable, having strong anti-herbivory properties. This is interesting because orang-utans put up with the bitter taste to chew the leaves, but do not ingest the leaves. Our biological analyses demonstrated that compounds in the leaves inhibit TNFα-induced inflammatory cytokines E-selectin, ICAM-1, VCAM-1 and IL-6 production, and therefore act as an anti-inflammatory agent. Thus, the rubbing of this soapy substance onto their limbs can provide anti-inflammatory benefits to the orang-utan. It also supports the reasons given by local indigenous people for their use of this plant.

In recent studies, steroidal saponins have been found in many species of *Dracaena*, including taxa of the morphologically-distinct but biogeographically-scattered dragon-tree species^26^. Secondary metabolites present in dragon-tree species have been suggested to confer antimicrobial, antiviral, analgesic and antioxidant activity^26^. Steroidal saponins have also been found in the palaeotropical mesophytic/evergreen forest species of *Dracaena* ^27,28^. Beneficial effects have been suggested in experimental animals, including anti-inflammatory activity^29^ based on steroids derived from *Dracaena mannii* (Baker) and wound healing^30^. Other species with anti-inflammatory and analgesic effects include *D. ombet* Heuglin ex Kotschy & Peyr^31^. Potential beneficial effects through cytotoxic and, hence, anticarcinogenic activity in saponins from *D. draco* have also been proposed^26^. Thus, there is much evidence for the medicinal use of many *Dracaena* species.

In Sabangau, it has been primarily adult females (9 out of 10 cases) observed performing this behaviour. One possible explanation for this may be the extra weight added by carrying offspring for females when climbing, and may also explain why they concentrate mainly on their arms when fur-rubbing. The fact that local people use the crushed leaves for sore muscles and joints further supports the concept that orang-utans would use it to treat similar problems. Local indigenous people in Borneo, for example, use it to treat pain in their arms after a stroke, for muscular pain, and for sore bones and swellings^19^. We conclude that the presence of anti-inflammatory properties in *D. cantleyi*, and the specific way in which this plant is used, make a compelling argument that orang-utans are practicing a form of self-medication. This finding is also important for studies of ethno-medicine, as it is known that indigenous communities obtain knowledge of medicinal plants through observing their use by sick animals^32^.

## Materials and Methods

### Study site

We carried out field research in the Sabangau Peat-swamp forest (600 km^2^) as part of the OUTROP – CIMTROP multi-disciplinary research project within the LAHG (*Laboratorium Alam Hutan Gambut*: *Natural Laboratory for the Study of Peat Swamp Forest*), a 50 km^2^ area in the Northern Sabangau Catchment. The LAHG was designated for the purpose of scientific research in 1997, and is managed by the Indonesian Research and Conservation Organisation CIMTROP (the Centre for International Co-operation in Management of Tropical Peatland). This forest is a protected tropical peat-swamp forest in the southern area of the Central Kalimantan province in Indonesia. The base camp is located 20 km southwest of Palangkaraya, the provincial capital.

### Plant identification and morphology

This species of *Dracaena* was identified from samples collected after each orang-utan performed the fur-rubbing act. Photographs and dried specimens including both flowering and fruiting parts of the plant were used to identify this species as *Dracaena cantleyi* by botanists at the herbaria in Palangkaraya, in Bogor, Indonesia and at Kew Gardens in the United Kingdom.

This species forms a woody shrub or small tree. It is part of Asparagaceae subfamily Nolinoideae. It is distributed throughout Borneo to Peninsular Malaysia^4^, where it is sometimes know under the illegitimate name *D. aurantiaca* (Baker) Wall. ex Hook.f. As is typical for the genus, it produces leaves in rosettes on woody stems. Leaf shape in *D. cantleyi* is rather variable, and the leaf blades are sometimes variegated with circular to elliptic, discrete to contiguous paler markings. The systematics of this genus have not been studied in tropical South-East Asia since Ridley’s era (1924), but it is suggested that *D. sara’wakensis* (W.W.Sm.) Jankalski from Borneo is a conspecific of *D. cantleyi* Baker based on its morphology. *D. cantleyi* is thought to be relatively infrequent in Central Kalimantan.

### Biological activities

#### Extract preparation

The dried plant material was homogenised to a fine powder in liquid nitrogen. Portions of the ground material (0.35 g) were then extracted separately in 35 ml of water, methanol and/or methanol:tetrahydrofurane (MeOH:THF, 1:1). After 16 hours (overnight) extraction at −20 °C, the resulting homogenates were centrifuged (15,000 rpm, 4 °C, 20 min). The sediments were re-extracted for 1 hour in the same way and then centrifuged. These two supernatants were pooled and dried in vaccuo at 35 °C, then dissolved in 200 μl of methanol and 800 μl of 0.1 M Tris buffer (pH 7.2).

#### Cell culture

Human Umbilical Vein Endothelial Cells (HUVEC) were cultured in ECGM medium (Endothelial Cell Growth Medium, Provitro, Berlin, Germany) and supplemented with 10% fetal bovine serum (Sigma Aldrich, Munich, Germany). Cells were maintained under standard cell culture conditions at 37 °C and 5% CO_2_ in a humid environment. Cells were subcultured two or three times a week using the standard trypsinization procedure. Calcein AM was obtained from Molecular Probes (Life Technologies, Carlsbad, CA, USA). HUVECs were a kind gift from Prof Jitka Ulrichová (Medical Faculty, Palacky University, Olomouc).

#### ELAM

CD62E (E-selectin, ELAM)-induction assays: Each well of the 96-well plates were coated with gelatine by applying 200 µl of 1.0% gelatine for 10 minutes at room temperature. Outer wells (A1-A12, H1-H12, 1-H1 and A12-H12) contained only 200 µl/well medium and served as an evaporation barrier. 1 × 10^4^ HUVECs were seeded in each of the other wells in a 200 µl medium and grown for 48 hours to optimal confluence. Increasing concentrations of extracts were then added to the HUVEC-containing wells in triplicates, and the cells were incubated for 30 minutes, after which 10 ng/ml TNFα was added per well to stimulate NF-κB, and thus ELAM. After another 4 hours of incubation, the levels of ELAM in each of the HUVEC-containing wells were determined by enzyme-linked activity assays (ELISAs) as described below.

Cell-surface ELISA ELAM: Cells were washed once with phosphate buffered saline (PBS) and fixed with 0.1% glutaraldehyde (Sigma-Aldrich, Munich, Germany) for 15 minutes at room temperature. Then, cells were washed three times with 200 µl per well PBS/0.05% Tween 20, blocked with 200 µl/well 5% bovine serum albumin (BSA)/PBS for 1 hour, and washed again three times with 200 µl per well PBS/0.05% Tween 20. Then anti-ELAM-antibody (clone BBA-1, R&D Systems, Minneapolis, MN, USA) diluted 1:5000 in 0.1% BSA/PBS (100 µl per well) was added for 1 hour at room temperature and washed thereafter five times with 200 µl per well PBS/0.05% Tween 20. Subsequently, goat anti-mouse horseradish-peroxidase (HRP) antibody (Sigma-Aldrich, Munich, Germany) diluted 1:10000 in 0.1% BSA/PBS (100 µl per well) was applied and the cells were incubated for 1 hour in a dark at room temperature and, after decanting, washed five times with 200 µl per well PBS/0.05% Tween 20. The HRP-activity of the cells in each of the wells was estimated using Fast-OPD (o-phenylenediamine dihydrochloride) (Sigma-Aldrich, Munich, Germany) assay and absorbance was measured at OD_492nm_ in a vertical spectrophotometer.

Cytotoxicity testing: For the ELAM expression assay, the toxicity of tested extracts was assessed in HUVECs by Calcein AM or sulforhodamine B (Sigma Aldrich, Munich, Germany) cytotoxicity assays in 96-well microtitre plates^33^. 20 µL portions of each of the extract concentrations were added in triplicate to the cells, which were then incubated at 37 °C in an atmosphere containing 5% CO_2_ for 4 hours. After this, Calcein AM solution (Molecular Probes, Invitrogen, Karlsruhe, Germany) was added for 1 hour, or sulforhodamine B after fixation with 50% trichloracetic acid, according to the manufacturer’s instructions. The fluorescence of viable cells stained with Calcein was quantified using a Fluoroskan Ascent instrument (Labsystems, Vantaa, Finland) reader and on the basis of triplicate experiments the cytotoxic concentrations were calculated. The absorbance of total protein mass related to the cell number stained with sulforhodamine B was measured using Tecan Infinite reader (Tecan Group, Männedorf, Switzerland).

#### ICAM-1, IL-6

Sandwich CD54 (ICAM-1) and IL-6 ELISAs: HUVEC cells were seeded into 24 well-plates and treated immediately with tested extracts for 30 minutes. MeOH/Tris was used as a vehicle for controls and TNFα (10 ng/ml) as inflammation stimuli. After 24 hours of treatment, culture medium was removed and collected into microfuge tube. Concentrations of cytokines (IL-6, ICAM-1) produced by cells into the medium was assessed using sandwich ELISA kits (PeproTech, Rocky Hill, NJ, USA). 96 well-plates for ELISA (Nunc, Thermo Fisher Scientific, Rockford, USA) were coated with capture antibody. After overnight incubation, plates were washed with 0.05% Tween in PBS and blocked with 1% BSA in PBS (Sigma-Aldrich, Munich, Germany) for 1 hour. Subsequently, plates were washed with PBS with Tween (0.05%) and standard or sample was added for 2 h. After washing, detection antibody was applied for another 2 hours. Avidin-HRP conjugate was incubated for 30 minutes and thereafter HRP activity was estimated by ABTS substrate (Sigma-Aldrich, Munich, Germany) and absorbance was measured at OD_405nm_ in a multimode plate reader (Infinite 200 PRO, Tecan Group, Männedorf, Switzerland).

#### ICAM-1, VCAM-1

Flow cytometric analysis of cell surface adhesion proteins (ICAM-1, VCAM-1): Treated HUVECs were trypsinized, seeded in 100-mm culture dishes, and immediately incubated with the test extracts. After 30 minutes, TNFα (10 ng/ml) was used as an inflammation stimulus. After 24 hours of incubation, the cells were again detached with trypsin, washed, fixed (4% formaldehyde), and stained with anti-ICAM-1 or anti-VCAM-1 antibody labelled with FITC (Becton Dickinson, NJ, USA) in PBS. Signal were then assessed with a flow cytometer (FACS Verse^TM^, Becton Dickinson, NJ, USA). In each experiment, the fluorescence of cells exposed to all treatments was expressed relative to the mean fluorescence of cells treated with TNFα alone (set as 100%), and changes in the expression of ICAM-1 or VCAM-1 on the cells’ surfaces were expressed in terms of relative changes in the mean (logarithmic) index of fluorescence intensity using BD FACSuite software (Becton Dickinson, NJ, USA). At least three different sets of experiments were performed in triplicates.

### Data Availability

Data for these analyses are available from the corresponding author.
